# The DEVA trial: protocol for a randomised controlled trial of dequalinium chloride versus usual care antibiotics for the treatment of bacterial vaginosis

**DOI:** 10.1186/s13063-022-06954-x

**Published:** 2022-12-21

**Authors:** Rebecca Haydock, Trish Hepburn, Jonathan Ross, Jane Daniels, Clare Brittain, Louise Jackson, Mara Ozolins, Janet Wilson

**Affiliations:** 1 Nottingham Clinical Trials Unit (NCTU), Applied Health Research Building, University of Nottingham, University Park, Nottingham, NG7 2RD UK; 2 Department of GU Medicine, University Hospitals Birmingham NHS Foundation Trust, Whittall Street Clinic, Whittall Street, Birmingham, B4 6DH UK; 3 Health Economics Unit, Institute of Applied Health Research, College of Medical and Dental Sciences, University of Birmingham, Edgbaston, Birmingham, B15 2TT UK; 4 GU Medicine Service, Leeds Teaching Hospitals NHS Trust, Leeds General Infirmary, Great George Street, Leeds, LS1 3EX UK

**Keywords:** Clinical trial, Randomised controlled trial, Protocol, Bacterial vaginosis, Antiseptic, Pessary, Metronidazole, Dequalinium

## Abstract

**Background:**

Bacterial vaginosis (BV) is the most common cause of vaginal discharge in women of reproductive age, and it is estimated that up to a third of women will experience it at some point in their lives. BV produces an offensive vaginal odour and it is associated with serious sequelae. The most frequently prescribed treatment for BV in the UK is 7-day oral metronidazole but recurrences are common following it. Dequalinium chloride (Fluomizin©) is an anti-infective, antiseptic agent administered as a vaginal tablet. Small studies have shown this to be an effective alternative to antibiotics as a BV treatment. This trial aims to investigate whether dequalinium is as effective as current antibiotic treatments for the treatment of BV 1 month after treatment start.

**Methods:**

DEVA is a multi-centre, randomised, open-label, parallel group, non-inferiority trial of dequalinium chloride versus usual care antibiotics for the treatment of BV. Recruitment will take place in 15 GUM clinics in the UK with Leeds Sexual Health also managing remote recruitment via the trial website. Women will be randomised 1:1 to receive dequalinium or usual care antibiotics. The primary outcome is to determine if the proportion of women reporting resolution of BV symptoms 4 weeks after treatment (without the need for additional treatment) is not worse in women treated with dequalinium chloride compared to usual care antibiotics. Questionnaire follow-up will take place 4 and 12 weeks after starting treatment, and remotely recruited patients will also provide a week 4 BV vaginal smear. The sample size is 904.

**Discussion:**

This trial will provide high-quality evidence on the use of dequalinium chloride as a BV treatment, which could result in patients reducing the number of antibiotics they take.

**Trial registration:**

ISRCTN ISRCTN91800263. Prospectively registered on 20 January 2020.

## Administrative information

Note: the numbers in curly brackets in this protocol refer to SPIRIT checklist item numbers. The order of the items has been modified to group similar items (see http://www.equator-network.org/reporting-guidelines/spirit-2013-statement-defining-standard-protocol-items-for-clinical-trials/).

**Table:** 

Title {1}	The DEVA trial: protocol for a randomised controlled trial of dequalinium versus usual care antibiotics for the treatment of bacterial vaginosis
Trial registration {2a and 2b}	ISRCTN, ISRCTN91800263 prospectively registered on 20 January 2020
Protocol version {3}	2.0 26 March 2021
Funding {4}	National Institute of Health Research (NIHR) [Health Technology Assessment programme (project reference 17/65/03)].
Author details {5a}	^1^Nottingham Clinical Trials Unit (NCTU), Applied Health Research Building, University of Nottingham, University Park, Nottingham NG7 2RD, UK. ^2^Department of GU Medicine, University Hospitals Birmingham NHS Foundation Trust, Whittall Street Clinic, Whittall Street, Birmingham B4 6DH, UK. ^3^Health Economics Unit, Institute of Applied Health Research, College of Medical and Dental Sciences, University of Birmingham, Edgbaston, Birmingham B15 2TT, UK. ^4^GU Medicine Service, Leeds Teaching Hospitals NHS Trust, Leeds General Infirmary, Great George Street, Leeds LS1 3EX.
Name and contact information for the trial sponsor {5b}	Leeds Teaching Hospitals NHS Trust LTHT.ResearchInformatics@nhs.net
Role of sponsor {5c}	The views expressed are those of the author(s) and not necessarily those of the Leeds teaching Hospitals NHS Trust, NIHR or the Department of Health and Social Care. The Sponsor and funder had no role in the design of this study and will have no role in the analysis, interpretation of data or decision to submit results in the future.

## Introduction

### Background and rationale {6a}

#### Background

Bacterial vaginosis (BV) is the most common cause of vaginal discharge in women of reproductive age. In 2019, 85,459 women were diagnosed with BV in English genitourinary medicine (GUM) clinics [1] (REF https://view.officeapps.live.com/op/view.aspx?src=https%3A%2F%2Fassets.publishing.service.gov.uk%2Fgovernment%2Fuploads%2Fsystem%2Fuploads%2Fattachment_data%2Ffile%2F1056731%2F2020_Table_4_All_STI_diagnoses_and_services_by_gender_and_sexual_risk_updated.ods&wdOrigin=BROWSELINK) and 15% of pregnant women attending an antenatal clinic have been found to have BV [2]. It is estimated that up to a third of women will get it at some time in their lives.

BV produces an offensive vaginal odour and is associated with serious sequelae [3]. It increases the acquisition of sexually transmitted infections (STIs) [4] and HIV [5] and the transmission of HIV [6]. It is associated with miscarriage, preterm birth [7] and pelvic inflammatory disease which causes infertility. [8]

The most frequently used treatment for BV in the UK is 7-day oral metronidazole. [3] This has microbiological resolution rates of 61–94% 1 month after treatment. [9] Recurrences are common, occurring in 23%, 43% and 52% of women at 1, 3 and 6 months post-treatment with metronidazole [10]. There are other antibiotic treatment regimens recommended in national guidelines using intravaginal metronidazole, intravaginal or oral clindamycin or oral tinidazole but the failure rates are similar [11]. Recurrent BV has a severe impact on quality of life [12] and vulvovaginal candidiasis often occurs following antibiotic treatment for BV. [13]

Dequalinium chloride (currently available under the brand name Fluomizin®) is an anti-infective and antiseptic agent belonging to the class of quaternary ammonium compounds. It is a surface-active substance and the primary mechanism of action is an increase in bacterial cell permeability leading to loss of enzyme activity, resulting in cell death. Dequalinium chloride exhibits rapid bactericidal activity and in the form of vaginal tablets exerts its action locally within the vagina. It is administered as a 10-mg vaginal tablet daily for 6 days. Advantages of dequalinium chloride in comparison to oral antibiotics used to treat bacterial vaginosis include broad antimicrobial spectrum, lower risk of development of resistance, achieving a high concentration of the substance at the infection site and minimal systemic exposure. [14] Dequalinium chloride was licensed for use in the UK in June 2015 and is currently the only antiseptic licensed for the treatment of BV in the UK. [15]

There is only one published randomised control trial (RCT) to date comparing dequalinium chloride with a guideline-recommended treatment, vaginal clindamycin cream. [16] This was a single-blinded, randomised trial in 15 sites and included 321 women. Women were randomised to either vaginal dequalinium chloride tablets or vaginal clindamycin cream. Follow-up visits were 1 week and 1 month after treatment. The trial showed a dequalinium chloride clinical cure rate, based on Amsel’s criteria, of 79.5% at 30 days but it did not include patient-centred outcomes and there was no longer-term follow-up to assess recurrences [16]. In an additional RCT comparing dequalinium chloride to povidone-iodine (which is not licensed for BV treatment and is not a guideline-recommended BV treatment), 78.3% of women treated with dequalinium chloride had BV resolution at 30 days. [17] However, no previous RCT has compared dequalinium chloride to the most commonly used antibiotics prescribed to treat BV.

#### Trial rationale

Alternative treatments for BV are clearly needed. In a qualitative study of women who had experienced BV, most were dissatisfied with current treatments. Although antibiotic treatment is effective at the time of treatment, many women felt frustrated and distressed at having BV recur, often quite quickly after treatment. Most women disliked taking antibiotics, especially on a regular basis, and felt frustrated at the lack of alternative effective treatments. [18] Identifying novel and effective treatments will improve the care and wellbeing of women experiencing BV and reduce their complications from it. Fewer return visits, and reduced complications, due to less frequently recurrent BV, also have the potential to reduce health service costs.

#### Justification for participant population

The target population is women with symptoms of BV with confirmation of BV on microscopy examination of a sample of vaginal discharge with the presence of vaginal odour and including those with either first or recurrent episodes. The trial is inclusive with primarily safety issues (e.g. allergies to dequalinium chloride or usual care antibiotics) or a clinical requirement for antibiotic therapy and/or the use of other intravaginal therapies, being exclusion criteria. The UK guideline for the management of bacterial vaginosis recommends that symptomatic pregnant women should be treated in the same way to non-pregnant women [3]. Therefore, pregnant women with an ongoing pregnancy who are not planning to seek a termination of pregnancy are eligible for inclusion within the trial. Also, unlike many other BV treatment trials, women living with HIV are eligible for inclusion.

## Objectives {7}

The primary objective is to determine if the proportion of women reporting resolution of BV symptoms 4 weeks after treatment is not worse in women treated with dequalinium chloride compared to usual care antibiotics, without the need for additional treatment**.**

## Trial design {8}

This is a phase IV, multi-centre, randomised, open-label, parallel group, non-inferiority trial with equal allocation (1:1) to receive either dequalinium chloride (intervention) or usual care antibiotics (control). Women will be recruited either face-to-face or remotely from a minimum of 15 sexual health clinics in the UK or following initial self-referral and completion of an expression of interest (EOI) form via the DEVA trial website (www.devastudy.ac.uk). The participant information sheet and animations to support the trial documentation can be found on the trial website. An embedded Study Within a Trial (SWAT) investigating the timing of reminder communications has been built into the trial design. Details of the SWAT can be found on the SWAT repository 118 [19] and will be reported separately.

## Methods: participants, interventions and outcomes

### Study setting {9}

The trial allows for patients to be identified and recruited into the trial by recruiting sexual health clinics or via the trial website, through one of three recruitment pathways. Recruitment was adapted from the original design to take into consideration changes that have been made to clinical services in response to the COVID-19 pandemic.

#### Pathway 1 (in-clinic consultation)

Patients attending in-person clinic consultations at participating recruitment clinics will be screened, consented and after providing a vaginal swab for microscopic confirmation of BV (confirmed by site staff, by following local procedures) be prescribed their randomly allocated treatment during their clinical consultation.

#### Pathway 2 (remote clinic consultation)

Patients with suspected BV will be identified locally and will have a remote consultation with a member of the clinical team where the trial will be discussed. The participant information sheet (PIS) will be provided and informed consent form (ICF) completed by the patient and investigator remotely. Each consented patient will be posted a vaginal swab kit to take two swabs—a BV smear swab to assess eligibility and a swab for STI screening (*Neisseria gonorrhoeae*, *Chlamydia trachomatis* and *Trichomonas vaginalis* testing using nucleic acid amplification tests). Patient-taken swab samples will be posted to the team at Leeds Sexual Health (LSH) for BV microscopy (using Nugent’s scoring) to confirm eligibility. LSH will report these results back to the recruiting site. The recruiting site will conduct a second remote consultation with the patient and those with BV on microscopy who want to participate will complete a final eligibility assessment and be randomised during the consultation. Trial medication will be dispensed and posted immediately by the site following randomisation, and the team at LSH will post out a week 4 vaginal BV smear sample kit.

#### Pathway 3

Patients with symptoms of BV will self-refer using an expression of interest (EOI) form on the trial website (www.devastudy.ac.uk). Patients who pass the EOI webform pre-screen will be contacted by a member of the DEVA research team at LSH, who will manage the care of all patients identified via the website. During the initial call, the team will assess whether the patient is eligible and interested in participating in the trial; those who are will be sent the PIS and informed consent form (ICF) electronically. Each consented patient will be posted a vaginal swab kit to take two swabs, a BV smear for eligibility and a swab for STI screening. Patient-taken swab samples will be returned to the team at LSH to confirm BV status and eligibility for the trial. Those with BV on microscopy who want to participate will complete final eligibility assessments and be randomised during a second consultation. Trial medication and the week 4 vaginal sample kit will be dispensed and posted to the patient by LSH.

### Eligibility criteria {10}

To be eligible for participation in the trial, women must meet the following eligibility criteria.

#### Inclusion criteria

- Cis-women aged 16 years and over
- Diagnosis of BV confirmed by microscopy with symptoms of vaginal odour plus or minus vaginal discharge requiring treatment with usual care antibiotics (as recommended in the BASHH BV treatment guideline)
- Willing to use either intravaginal dequalinium chloride tablets (pessaries) or the clinician-selected usual care antibiotic BV treatment
- Willing to avoid vaginal sex whilst taking/using trial treatment
- Willing to avoid vaginal douching whilst taking/using trial treatment
- Willing to complete follow-up questionnaires in English
- Able to provide written informed consent

#### Exclusion criteria

- Contra-indications or allergy to dequalinium chloride or clinician-selected usual care antibiotic BV treatment
- Use of oral antibiotics concurrently, within the 14 days prior to randomisation or planned use over the 14 days from randomisation
- Use of intravaginal therapies (including vaginal douching) concurrently, within the 14 days prior to randomisation or planned use over the 14 days from randomisation
- Pregnant women who are seeking a termination
- Previous participation in this trial
- Unwilling to provide GP information (only applicable for pregnant women or women who become pregnant in the first 4 weeks of the trial)
- Resident outside the UK (only applicable if shipment of trial medication and sample kits is required)

### Who will take informed consent? {26a}

Consent for the trial will be obtained in writing either using a paper or electronic consent form. All patients, regardless of the pathway, will be given the opportunity to ask questions prior to completing their consent form. Consent will be obtained by a member of the research team at the local recruiting clinic or LSH, in accordance with the delegation of responsibilities authorised by the principal investigator. Consent can be taken by a doctor or research nurse. Prior to randomisation, each patient must have their eligibility for the trial assessed and confirmed by a doctor and documented on the eligibility checklist.

### Additional consent provisions for collection and use of participant data and biological specimens {26b}

Consent forms include optional consent for receiving trial results by email at the end of the study, the sharing of patient details with a third party for coordination of trial reminders (by SMS) and for those recruited remotely, the option to provide an additional vaginal sample at baseline and week 4 for future research studying vaginal microbiota.

## Interventions

### Explanation for the choice of comparators {6b}

Control treatment includes any UK guideline-recommended/alternative oral or topical antibiotic treatment for BV. This makes the trial more generalisable as it will allow a greater population of patients to be included (e.g. those with contra-indications to certain antibiotics and those who do not wish to take oral antibiotics) and be responsive to sporadic shortages of particular antibiotics.

Clinicians will select the control treatment prior to randomisation, after discussing the treatment options with the patient. If the patient is randomised to the control arm, they will receive this predetermined BV treatment.

### Intervention description {11a}

Women allocated to the dequalinium chloride arm (intervention) will be asked to follow the UK-licensed treatment course of one 10-mg vaginal tablet for 6 nights.

### Criteria for discontinuing or modifying allocated interventions {11b}

Participants will be encouraged to take the full course of their allocated medication. Modification or discontinuation of the allocated intervention is at the discretion of the treating clinician.

### Strategies to improve adherence to interventions {11c}

As there are no visits following randomisation, participants will be given information on how to take their treatment during their consultation, with additional information provided in medication labelling on the treatment box and in a medication patient leaflet as per standard clinical practice. Reminder messages (email and SMS) will be sent to participants to remind them that they are participating in the trial and to inform them when their trial questionnaires or samples are due to be returned. Adherence to treatment and questionnaire completion will be monitored monthly by the Trial Management Group (TMG).

### Relevant concomitant care permitted or prohibited during the trial {11d}

Planned use of oral antibiotics or intravaginal therapies concurrently or for use 14 days following randomisation forms part of the exclusion criteria for the trial. Where additional medications are used as a treatment for BV participants will be asked to record the treatment and amount used on their follow-up questionnaires.

### Provisions for post-trial care {30}

There are no provisions for post-trial care. However, participants will be advised to seek care from a clinician if they do not experience resolution of their symptoms.

### Outcomes {12}

The primary outcome measure is participant-reported resolution of BV symptoms 4 weeks post-treatment start date without the need for additional treatment. Secondary outcome measures include time to participant-reported resolution of BV symptoms without the need for additional treatment, microscopic resolution of BV on microscopy at week 4 (without the need for additional treatment) as assessed by central laboratory analysis of participant-taken vaginal smears in a sub-group of participants and cost of BV treatment, including additional medication and healthcare usage relating to BV. A full list of outcome measures has been reported on the ISRCTN—https://www.isrctn.com/ISRCTNISRCTN91800263.

### Participant timeline {13}

Participants are recruited into the trial for a total of 12 weeks following randomisation. Baseline assessment randomisation and treatment dispensing will be performed by the recruiting centres, with follow-up questionnaires at weeks 4 and 12 coordinated by the NCTU and week 4 samples coordinated by LSH. The summary of trial procedures is detailed in Table 1.

**Table 1:** Summary of trial procedures by time point

	Baseline assessment (pre-randomisation)		Randomisation	Follow-up (time from randomisation)		
	Pathway 1	Pathways 2 and 3	Day 1	Day 14	Week 4	Week 12
**Procedure**						
**a** – Eligibility screen	**X**	**X**				
**b** – Informed consent	**X** (paper)	**X** (electronic)				
**c** – Baseline data collection	**X**	**X**				
**d** – Randomisation			**X**			
**e –** Prescribe usual care treatment	**X**	**X**				
**f –** Issue randomised trial treatment			**X** For remote pathways, ‘issue’ refers to the shipment of treatment to the participant			
**g** – Vaginal swab for STI testing	**X**	**X ~ ** Self-taken, posted by a participant				
**h** – Vaginal smear for microscopy	**X** (following local procedures)	**X** Self-taken, posted by a participant			**X** Remote only Self-taken, posted by participant	
**i –** Vaginal swab for future study of vaginal microbiota*		X* Self-taken, posted by a participant			X* Remote only Self-taken, posted by a participant	
**Interventions**						
**j** – Dequalinium chloride (intervention group)			**X** (days 1–6)			
**k** – Usual care antibiotics (control group)			**X** (duration of treatment in accordance with usual care)			
**Follow-up**						
**l –**Treatment adherence text message				**X**		
**m** – Participant questionnaire (online or postal based on preference)					**X**	**X**
**n – Pregnancy outcome**^**#**^			The outcome of participants pregnant at the time of randomisation or found to have conceived between randomisation and week 4 follow- up will be collected throughout the duration of the trial			

### Sample size {14}

Assuming 78% of participants have BV symptom resolution at 4 weeks post-treatment start date in both the control and intervention groups [9, 16, 17], a total sample size of 722 for analysis (361 in each group) will achieve 90% power to conclude non-inferiority with a lower confidence limit for the absolute risk difference of 10%, using a 1-sided significance level of 0.025. To allow for a loss of primary outcome data of up to 20%, the trial will recruit a total of 904 women. The 10% non-inferiority margin was acceptable to 68% of a sample of women attending two GUM clinics and 81% of clinicians experienced in treating BV.

### Recruitment {15}

To ensure the trial remains pragmatic and in line with sexual health services which are being provided following COVID-19, patients can join the trial via one of three recruitment pathways which are available—through a SHC recruitment centre (in-person or remote) or via patient self-referral on the trial website.

## Assignment of interventions: allocation

### Sequence generation {16a}

Participants will be assigned to treatment groups using a remote Internet-based randomisation system developed and maintained by the NCTU.

Treatment will be assigned using a minimisation algorithm balancing on the following factors:

- First episode or isolated recurrence of BV (no previous episodes in the past year) or recurrence (previous episode in the past year)
- A female sexual partner in the previous year, or not. [20]
- Method of contraception (none, barrier, intrauterine, hormonal; if more than one method is used, they will be prioritised as intrauterine, hormonal then barrier) [20–23]
- Whether they consent to provide self-taken samples for the sub-study at baseline and week 4, or not
- Recruitment site
- Recruitment pathway (1, 2 or 3)

The randomisation system is concealed and the minimisation algorithm will include a probabilistic element to allocation making prediction of the allocated group virtually impossible.

### Concealment mechanism {16b}

Allocation will be concealed to all trial staff using an automated web system operated by the NCTU. The control treatment will be determined and recorded by the clinician for each participant prior to randomisation.

### Implementation {16c}

An authorised member of the site research team will then log into the secure randomisation system and randomise the participant following completion of informed consent, confirmation of eligibility and the assignment of usual care treatment should the patient be randomised to the control treatment arm*.*

## Assignment of interventions: blinding

### Who will be blinded {17a}

Blinding of the participant and site research team is not possible due to the nature of the intervention; several different usual care antibiotics with different routes, formulations and duration will be used in the control arm.

Central laboratory staff performing BV microscopy and STI testing will be blinded to the participant’s treatment allocation. The chief investigator, deputy chief investigator and trial management team will be blinded to the treatment allocation of participants recruited in the trial. Trial and senior trial statisticians will be blinded to participants’ treatment allocation, including other data with the potential to lead to unblinding, until after the statistical analysis plan (SAP) is finalised and approved and the trial database (including randomisation system) is locked. Any data summaries and analyses which require knowledge of the treatment allocation, e.g. within the closed report for the Data Monitoring Committee (DMC), will be conducted by a statistician who is independent of the trial. Such summaries and analyses will be held in an area which is accessible only to the statistician(s) who are independent of the trial.

### Procedure for unblinding if needed {17b}

Site staff and participants are unblinded to treatment allocation so there is no procedure for emergency unblinding in place.

## Data collection and management

### Plans for assessment and collection of outcomes {18a}

Most of the outcomes are collected in the week 4 and 12 questionnaires, with the information obtained directly from the participants. Additionally, a text message will be sent at day 14 to those who consent, to collect treatment adherence data. In cases where participants do not respond to this, adherence data will be collected in the week 4 questionnaire.

For the participants recruited through pathways 2 and 3, the presence of BV 4 weeks after starting treatment will be assessed on microscopy of a self-taken vaginal smear.

### Plans to promote participant retention and complete follow-up {18b}

Strategies to minimise loss to follow-up will include using text message, email and phone reminders made to participants. Each questionnaire has a completion window to allow participants to complete the questionnaires even if they are unavailable at the 4- and 12-week time points. Participants may also be contacted by telephone by a member of the trial team in order to collect follow-up data if no responses are received.

A website has been created for the trial where updates on trial activity and relevant information relating to the trial and BV will be published. Additionally, accounts will be maintained across social media platforms to keep participants and potential participants informed of trial activity.

### Data management {19}

All trial baseline data will be collected by site staff and entered onto the trial bespoke Randomisation system and MACRO database. Both systems will be developed and maintained by the NCTU. Access to the database will be restricted and secure. Sites will be provided with paper workbooks to assist them with data collection. Missing or spurious data will be queried in a timely manner throughout the trial. Follow-up data will be collected via two participant-completed questionnaires, at 4 and 12 weeks post-treatment start date, and these will be sent either electronically via a secure link or as a paper questionnaire sent to the participant’s address, with a pre-paid return envelope. Identifiable information about participants (such as address data for questionnaires and samples) will be held in a separate database to the trial data with access restricted to those involved in follow-up only, as authorised by the CI. All access and data transactions within the databases will be logged with a full audit trail.

### Confidentiality {27}

Individual participant medical information obtained as a result of this trial is considered confidential and disclosure to third parties is prohibited with the exceptions noted in this protocol.

Participants’ contact details, including name, address, telephone/mobile number and email, will be shared between participating sites and NCTU for the purposes of issuing sample kits, questionnaires and electronic reminders (text/email) for the trial.

Minimal linked anonymised data (participation identification code, initials and date of birth), used for labelling of laboratory samples, will also be shared with Leeds Teaching Hospitals NHS Trust.

Any personal data will be held in a secure database using encryption, with restricted password-protected access. Only appropriate members of the participating site team and NCTU research team will have access to these data.

Participant confidentiality will be further ensured by utilising identification code numbers to correspond to treatment data in computer files.

### Plans for collection, laboratory evaluation and storage of biological specimens for genetic or molecular analysis in this trial/future use {33}

A sub-group of patients screened remotely (pathways 2 and 3) will be invited to participate in the sub-study which involves consenting to and providing an additional self-taken vaginal microbiota swab at baseline and 4 weeks post-treatment start date. The consent for this will be optional, and participants who do not give their consent will not be precluded from participating in the trial. On receipt, these samples will be frozen at − 70 °C and stored at Leeds Teaching Hospital NHS Trust. These samples will be used for future analysis of the vaginal microbiota to assess any changes between baseline and week 4. The analysis of these will be the subject of a future grant application and further ethical approval and only the research team will have access to the samples whilst in storage.

## Statistical methods

### Statistical methods for primary and secondary outcomes {20a}

The analysis and reporting of the trial will be in accordance with CONSORT guidelines. A full statistical analysis plan (SAP) will be developed by the blinded trial statistician and agreed prior to database lock and release of treatment allocations.

The primary approach to the primary comparative analysis will be to analyse as randomised without imputation of missing data unless the proportion of missing data is greater than anticipated (i.e. greater than 20%) with due emphasis being placed on the confidence intervals for the between-arm comparisons. It is expected that adherence with trial treatment will be high. However, if it is lower than expected, a complier average causal effect (CACE) analysis will be performed to check the stability of the conclusions. Compliers will be those reporting the use of at least 75% of the treatment. The SAP will provide details of circumstances where a CACE analysis should be performed.

Continuous variables will be summarised, dependent on distribution, in terms of the mean, standard deviation, median, lower and upper quartiles, minimum, maximum and number of observations. Categorical variables will be summarised in terms of frequency counts and percentages. Descriptive statistics of demographic and clinical measures will be used to assess balance between the randomised arms at baseline, but no formal statistical comparisons will be made.

The evaluation of the primary outcome will be performed using a mixed effects model for the binary outcome that includes factors used in the minimisation (first/isolated episode or recurrent BV, female sexual partner in the previous year, method of contraception, pathway; the inclusion of site will be dependent on model convergence). If this model does not converge, a generalised estimation model with site as a panel variable may be used instead. The primary effectiveness parameter comparing dequalinium chloride with the usual care arm will be the risk difference in the proportion of participants who report resolution of symptoms 4 weeks after treatment for BV with no additional treatment, along with the 2-sided 95% confidence interval. Dequalinium chloride will be regarded as non-inferior to usual care antibiotics if the lower bound of this 2-sided 95% confidence interval for the risk difference (dequalinium chloride minus usual care antibiotics) is − 10% or greater (e.g. a lower bound of 9% would infer non-inferiority), i.e. dequalinium chloride is not likely to be worse than usual care antibiotics by more than 10%. Secondary outcomes with the exception of side effect data will be analysed using appropriate regression models including minimisation variables and baseline values of that outcome if measured. The analyses of secondary outcomes will be considered supportive to the primary and estimates and *p*-values (where presented) should be interpreted in this light.

Side effect data will be summarised using descriptive statistics according to the treatment the participant actually received, irrespective of randomisation.

The economic analysis will compare the costs associated with dequalinium chloride with usual care antibiotics. The economic evaluation will use only data collected within the trial and so estimates of costs and outcomes will therefore relate only to the initial period and assessment at week 4 and week 12. The primary analysis will be based on cost per patient with resolved symptoms at week 4 (as per the trial primary outcome), with a secondary analysis of cost per patient without recurrence of symptoms at week 12.

### Interim analyses {21b}

There will be no planned interim effectiveness analyses. However, an internal pilot phase has been built into the trial to allow a feasibility assessment which will examine recruitment, retention and adherence. Stop–go criteria 9 months after the first participant has been randomised will be used to determine the progression of the trial. Recruitment will be measured against the overall recruitment target. Retention will be determined by provision of primary outcome data. Adherence level will be measured using simple categories asking how much of the trial medication the participant used/took (high > 75% used/taken, more than half (50–75% used/taken), less than half (1–49% used/taken), from both treatment arms.

The SWAT will also undergo its own interim analysis which will be detailed in the SAP for the SWAT.

### Methods for additional analyses (e.g. sub-group analyses) {20b}

The trial is powered to detect overall differences between groups rather than interactions; therefore, any sub-group analyses will be regarded as exploratory. Planned sub-group analyses will be pre-specified in the SAP.

### Methods in analysis to handle protocol non-adherence and any statistical methods to handle missing data {20c}

Several strategies to investigate the effect of missing primary outcome data will be undertaken as sensitivity analyses, including the use of multiple imputation with chained equations and minimisation variables as covariates. Where the proportion of missing primary outcome data is greater than 20%, the primary analysis will use multiple imputation. More information on methods will be included within the SAP.

### Plans to give access to the full protocol, participant-level data and statistical code {31c}

De-identified participant data will be made available, upon request, in accordance with the NCTU standard operating procedures following the publication of the trial results.

## Oversight and monitoring

### Composition of the coordinating centre and trial steering committee {5d}

Initially, on-site monitoring of the BV eligibility and baseline STI sample results for recruited participants will take place at least annually at the lead site (LSH) but this may be increased/reduced as deemed necessary. Other than this, on-site monitoring will not be conducted routinely throughout the trial. Central monitoring of all trial data (across recruiting centres and participant-reported data) will be undertaken and used to assess whether sites have met any of the monitoring triggers detailed in the trial monitoring plan. The TMG will review ongoing trial data monthly and discuss the requirement for site visits if clinics meet monitoring triggers.

### Composition of the data monitoring committee, its role and reporting structure {21a}

An independent Data Monitoring Committee (DMC) will review unblinded trial data, including safety data, on a minimum annual basis. The DMC will operate in accordance with the trial-specific charter. Independent oversight will be provided by the Trial Steering Committee (TSC) who will meet at the same frequency as the DMC. Additional meetings may be called at the request of the DMC or TSC chair.

### Adverse event reporting and harms {22}

All medications used in the trial are UK-licensed drugs being administered within their licensed indications with well-characterised safety profiles. To provide secondary outcome data about the adverse events associated with the use of dequalinium chloride or any of the usual care antibiotics, only specified adverse reactions experienced during treatment will be collected on the week 4 follow-up questionnaire.

Serious adverse events (SAEs) are not anticipated in this trial; however, we will collect details of SAEs experienced via the week 4 follow-up questionnaire. Any participant who indicates they have been to the hospital in relation to their BV or BV treatment will be contacted to assess the event against the SAE reporting criteria.

### Frequency and plans for auditing trial conduct {23}

An ongoing review of trial conduct will be undertaken by the trial management team as per the trial monitoring plan. Audits may be carried out by the sponsor or NCTU quality assurance teams in accordance with their local auditing plans.

### Plans for communicating important protocol amendments to relevant parties (e.g. trial participants, ethical committees) {25}

Any amendments made to the trial protocol will undergo review and approval by the sponsor, MHRA, Research Ethics Committee and Health Research Authority prior to implementation. Updated versions of the protocol will be shared with recruiting centres via email and uploaded to the trial website. Any substantial amendments made will also be communicated to the trial registry.

## Dissemination plans {31a}

The results of the trial will be published in the NIHR Journals Library and will be submitted for publication in a peer-reviewed journal. Participants who provided consent to receive the trial results will be emailed a research result summary following publication and this will also be made available on the trial website.

## Discussion

To our knowledge, this is the first randomised trial in patients with confirmed BV where they can screen and participate without the need to attend any in-person consultations with a healthcare provider.

Since the start of the COVID-19 pandemic, one of the challenges we faced was how to design the trial to ensure screening and recruitment could continue to take place despite national lockdowns which may prevent patients from accessing in-person clinical care. To address this, we implemented the two remote recruitment pathways. The introduction of a website recruitment pathway will improve the generalisability of the results for the trial as it opens up the trial to all women in the UK creating a much larger, more diverse group of potential participants, compared to those attending a local sexual health clinic. We hope that it will encourage women who may not feel comfortable visiting their GP or local sexual health clinic with their BV symptoms to participate as there is no in-person contact and all trial samples can be taken privately at home.

To support the identification and recruitment of patients, we have designed and launched an advertising campaign for the trial on social media and search engines. Our social media adverts are targeted to specific demographics using a range of different images of women who represent our target age groups and a diverse range of ethnicities. We will monitor the engagement of each of the adverts to see which are the most successful and whether this is impacting the demographic of the participants that we are recruiting.

A limitation to the trial design is the reliance of pathway 3 recruitment on the advertising campaign and overall success of publicising the trial. As women will not be introduced to the trial in the traditional format, during a consultation, it is important that we have full oversight and understanding of the advert engagement statistics and continue to develop campaigns in collaboration with our patient and public involvement reps to ensure the adverts remain relevant and appealing. Additionally, whilst we have implemented a number of different methods to keep participants engaged following recruitment, we are unsure whether the recruitment approach (in-clinic or remote) will impact the overall adherence to treatment and trial retention but we will monitor this closely.

The results from this trial will provide important information on the effectiveness of dequalinium chloride and usual care antibiotics on treating BV symptoms and provide us with valuable information on how to best manage BV patients and could potentially reduce the number of antibiotics prescribed to treat this condition.

## Trial status

At the time of the initial manuscript submission, 452 patients had been recruited. The first participant was recruited on 25 Aug 2021, and recruitment is planned to close on 31 December 2022 with a 3-month follow-up.

## Data Availability

Data sharing is not applicable to this article as no datasets were generated or analysed in preparation of this publication. De-identified participant data will be made available, upon request, in accordance with the NCTU standard operating procedures following the publication of the trial results.

## Abbreviations

BV: Bacterial vaginosis
CACE: Complier average causal effect
DMC: Data monitoring committee
EOI: Expression of interest
GUM: Genitourinary medicine
ICF: Informed consent form
NCTU: Nottingham Clinical Trials Unit
PIS: Patient information sheet
PPI: Patient and public involvement
RCT: Randomised control trial
SAE: Serious adverse event
SAP: Statistical analysis plan
STI: Sexually transmitted infection
SWAT: Study within a trial
TMG: Trial Management Group
TSC: Trial steering committee

## References

[1] Public Health England. Table 4: all STI diagnoses and services by gender and sexual risk, 2014 to 2018. https://www.gov.uk/government/statistics/sexually-transmitted-infections-stis-annual-data-tables.

[2] Hay PE, Lamont RF, Taylor-Robinson D, Morgan DJ, Ison C, Pearson J (1994). Abnormal bacterial colonisation of the genital tract and subsequent preterm delivery and late miscarriage. Br Med J.

[3] Clinical Effectiveness Group British Association for Sexual Health and HIV. UK national guideline for the management of bacterial vaginosis 2012. http://www.bashh.org/documents/4413.pdf. 10.1177/0956462414554435 25332225

[4] Brotman RM (2011). Vaginal microbiome and sexually transmitted infections: an epidemiologic perspective. J Clin Invest.

[5] Atashili J, Poole C, Ndumbe PM (2008). Bacterial vaginosis and HIV acquisition: a meta-analysis of published studies. AIDS.

[6] Cohen CR, Lingappa JR, Baeten JM (2012). Bacterial vaginosis associated with increased risk of female-to-male HIV-1 transmission: a prospective cohort analysis among African couples. PLoS Med.

[7] Leitich H, Bodner-Adler B, Brunbauer M (2003). Bacterial vaginosis as a risk factor for preterm delivery: a meta-analysis. Am J Obstet Gynecol.

[8] Haggerty CL, Totten PA, Tang G (2016). Identification of novel microbes associated with pelvic inflammatory disease and infertility. Sex Transm Infect.

[9] Joesoef MR, Schmid GP, Hillier SL (1999). Bacterial vaginosis: review of treatment options and potential clinical indications for therapy. Clin Infect Dis.

[10] Bradshaw CS, Morton AN, Hocking J (2006). High recurrence rates of bacterial vaginosis over the course of 12 months after oral metronidazole therapy and factors associated with recurrence. J Inf Dis.

[11] Sobel JD, Ferris D, Schwebke J, Peipert J (2006). Suppressive antibacterial therapy with 0.75% metronidazole vaginal gel to prevent recurrent bacterial vaginosis. Am J Obstet Gynecol.

[12] Oduyebo OO, Anorlu RI, Ogunsola FT. The effects of antimicrobial therapy on bacterial vaginosis in non-pregnant women. Cochrane Database Syst Rev. 2009;(3):CD006055. 10.1002/14651858.CD006055.pub2. 10.1002/14651858.CD006055.pub2 19588379

[13] Bilardi JE, Walker S, Temple-Smith M (2013). The burden of bacterial vaginosis: women’s experience of the physical, emotional, sexual and social impact of living with recurrent bacterial vaginosis. PLoS One.

[14] Mendling W, Weissenbacher ER, Gerber S (2016). Use of locally delivered dequalinium chloride in the treatment of vaginal infections: a review. Arch Gynecol Obstet.

[15] Dequalinium for bacterial vaginosis. Drug Ther Bull. 2017;55(5):54–7. 10.1136/dtb.2017.5.0478. 10.1136/dtb.2017.5.0478 28495833

[16] Weissenbacher ER, Donders G, Unzeitig V (2011). A comparison of dequalinium chloride vaginal tablets and clindamycin vaginal cream in the treatment of bacterial vaginosis: a single-blind, randomised clinical trial of efficacy and safety. Gynecol Obstet Invest.

[17] Petersen EE, Weissenbacher ER, Hengst P, Spitzbart H, Weise W, Wolff F (2002). Local treatment of vaginal infections of varying etiology with dequalinium chloride or povidone iodine. Arzneim Forsch/Drug Res.

[18] Bilardi J, Walker S, McNair R (2016). Women’s management of recurrent bacterial vaginosis and experiences of clinical care: a qualitative study. PLoS One.

[19] Hepburn T, Brittain C, Haydock R, et al. SWAT 118: timing of text message and email communication to improve questionnaire return rates. 2019. https://www.qub.ac.uk/sites/TheNorthernIrelandNetworkforTrialsMethodologyResearch/FileStore/Filetoupload,976326,en.pdf.

[20] Bradshaw CS, Walker J, Fairley CK, Chen MY, Tabrizi SN (2013). Prevalent and incident bacterial vaginosis are associated with sexual and contraceptive behaviours in young Australian women. PLoS One.

[21] Hutchinson KB, Kip KE, Ness RB (2007). Condon use and its association with bacterial vaginosis and bacterial vaginosis-associated vaginal microflora. Epidemiology.

[22] Achilles SL, Austin MN, Meyn LA, Mhlanga F, Chirenje ZM, Hillier SL. Impact of contraceptive initiation on vaginal microbiota. Am J Obstet Gynecol 2018: 622.e1–622.e10. 10.1016/j.ajog.2018.02.017. 10.1016/j.ajog.2018.02.017 PMC5990849 29505773

[23] Stewart CMW, Schoeman SA, Booth RA, Smith SD, Wilcox MH, Wilson JD (2012). Assessment of self taken swabs versus clinician taken swab cultures for diagnosing gonorrhoea in women: single centre, diagnostic accuracy study. BMJ.

